# Assessing service and treatment needs and barriers of youth who use illicit and non-medical prescription drugs in Northern Ontario, Canada

**DOI:** 10.1371/journal.pone.0225548

**Published:** 2019-12-05

**Authors:** Cayley Russell, Maria Neufeld, Pamela Sabioni, Thepikaa Varatharajan, Farihah Ali, Sarah Miles, Joanna Henderson, Benedikt Fischer, Jürgen Rehm

**Affiliations:** 1 Institute for Mental Health Policy Research, Centre for Addiction and Mental Health (CAMH), Toronto, ON, Canada; 2 Institut für Klinische Psychologie und Psychotherapie, Technische Universität Dresden, Dresden, Germany; 3 Translational Addiction Research Lab, Centre for Addiction and Mental Health (CAMH), Toronto, ON, Canada; 4 Department of Anesthesia and Pain Management, Toronto General Hospital, Toronto, ON, Canada; 5 Department of Psychiatry, University of Toronto, Toronto, ON, Canada; 6 The Margaret and Wallace McCain Centre for Child, Youth and Family Mental Health, Centre for Addiction and Mental Health, Toronto, ON, Canada; 7 Centre for Applied Research in Mental Health and Addiction (CARMHA), Faculty of Health Sciences, Simon Fraser University, Vancouver, Canada; 8 Faculty of Medical and Health Sciences, University of Auckland, Auckland, New Zealand; 9 Department of Psychiatry, Federal University of São Paulo (UNIFESP), São Paulo, Brazil; 10 Institute of Medical Science (IMS), University of Toronto, Toronto, ON, Canada; 11 Dalla Lana School of Public Health, University of Toronto, Toronto, ON, Canada; 12 Department of International Health Projects, Institute for Leadership and Health Management, I.M. Sechenov First Moscow State Medical University, Moscow, Russian Federation; University of Alberta, CANADA

## Abstract

**Background:**

Illicit drug use rates are high among Canadian youth, and are particularly pronounced in Northern Ontario. The availability and accessibility of effective substance use-related treatments and services are required to address this problem, especially among rural and remote Northern communities. In order to assess specific service and treatment needs, as well as barriers and deterrents to accessing and utilizing services and treatments for youth who use illicit drugs in Northern Ontario, we conducted the present study.

**Methods:**

This study utilized a mixed-methods design and incorporated a community-based participatory research approach. Questionnaires were administered in conjunction with audio-recorded semi-structured interviews and/or focus groups with youth (aged 14–25) who live in Northern Ontario and use illicit drugs. Interviews with ‘key informants’ who work with the youth in each community were also conducted. Between August and December 2017, the research team traveled to Northern Ontario communities and carried out data collection procedures.

**Results:**

A total of 102 youth and 35 key informants from eleven different Northern Ontario communities were interviewed. The most commonly used drugs were prescription opioids, cocaine and crack-cocaine. Most participants experienced problems related to their drug use, and reported ‘fair’ mental and physical health status. Qualitative analyses highlighted an overall lack of services; barriers to accessing treatment and services included lack of motivation, stigmatization, long wait-lists and transportation/mobility issues. Articulated needs revolved around the necessity of harm reduction-based services, low-threshold programs, specialized programming, and peer-based counselling.

**Conclusions:**

Although each community varied in terms of drug use behaviors and available services, an overall need for youth-specific, low-threshold services was identified. Information gathered from this study can be used to help inform rural and remote communities towards improving treatment and service system performance and provision.

## Introduction

Illicit drug use is a major public health concern worldwide, with an estimated 5.6% of the global population reporting use in 2016, and approximately 31 million of these users meeting requirements for a drug use disorder diagnosis [1]. Illicit drug use rates are commonly elevated among youth [2–4]. For example, in Canada, 20% of general population adolescents aged 15–19, and 35% of those aged 20–24, reported past-year use of illicit drugs in 2017 [4]. Moreover, the majority of people who use illicit drugs in Canada are between the ages of 15 to 24, and those who fall into this age category have been found to use drugs more heavily and in riskier ways compared to their older counterparts [5, 6].

Illicit drug use rates among youth in Canada vary across the provinces, with Ontario consistently showing high use rates among adolescents compared to many other provinces [7–9]. In particular, non-medical prescription opioid use among adolescents has been associated with a variety of adverse health outcomes, including fatal overdoses; there were 240 opioid-related overdose deaths among those aged 24 or younger in Ontario between 2013–2016, 92% of whom did not have an active opioid prescription [10].

Not only do drug use rates vary across the provinces, they also vary within [8, 9, 11]. For instance, significant regional differences in substance use rates among youth have been found in Ontario (e.g., between the province’s Local Integrated Health Networks (LHINs)) (see S1 Appendix), with higher rates of certain substances (e.g., alcohol) reported in Northern Ontario [8, 12]. Prescription opioid use in particular has been found significantly higher among youth in Northern Ontario; accordingly, in 2014, the rate of methadone maintenance treatment patients per 100,000 among youth (ages 15–24) was approximately 2-fold and 6-fold higher among the North East and North West LHIN, respectively, compared to the other LHINs in Ontario [13].

In order to address illicit and non-medical prescription drug use among youth in Northern Ontario, it is imperative that appropriate and culturally relevant treatment options and services are available [14–18]. There are a number of community-based addiction services in Northern Ontario, which include public, private, and not-for-profit organizations and services, as well as indigenous/culturally-based treatment centers [19, 20]. In terms of opioid agonist treatment for opioid use disorders, evidence suggests that the availability of these programs has recently increased in Ontario, particularly in rural and Northern areas where it had typically been underutilized [13, 21].

Distinct challenges in providing adequate treatment and service provision in rural and remote communities have been identified [22–26]. For instance, utilization of addiction services has been found lower in rural compared to urban areas [25–29], while access to care has been described as a major challenge since resources are concentrated in cities or larger communities and there are often shortages of healthcare providers [23, 25, 26, 30–32]. In 2017, youth under the age of 25 living in the North West LHIN had the highest rates of emergency department visits for addiction issues compared to the rest of Ontario [33], which suggests that there is a lack of access to, or awareness of community-based treatment options.

With this in mind, it is unclear whether or not the addiction-related services that exist in Northern Ontario are being accessed and utilized by the youth who need them, or whether or not the services are effectively addressing their needs. As such, gaining a thorough understanding of the service and treatment needs of youth who use drugs in Northern Ontario communities is essential for identifying and addressing lapses in current service availability and utilization. However, this information is lacking.

Thus, in order to explore the gaps and opportunities in service and treatment needs of youth who use illicit drugs in Northern Ontario, our research team conducted a study examining these issues utilizing a tailored mobile research lab. The research lab enabled the team to conduct data collection processes across multiple locations in a standardized manner, which was essential since Northern Ontario communities are extremely remote, hard to access and considerably dispersed from one another [34].

The present contribution sheds light on drug use patterns and drug-related health outcomes of youth who use illicit and non-medical prescription drugs in Northern Ontario. We examined needs, major barriers and deterrents, as well as gaps in terms of services and treatments for drug use among this unique population. This information can be used to better meet the needs of youth as well as provide suggestions for service system improvement in Northern Ontario as well as remote and/or rural communities more generally.

## Methods

The registered study protocol and further details on the methodology can be found in Varatharajan et al. (2018) [35]. The study was approved by the Centre for Addiction and Mental Health Research Ethics Board (REB# 133/2015).

### Aims and objectives

The objectives of this study were to identify any major gaps in service provision based on the articulated needs of the youth and key informants who reside in Northern Ontario communities, as well as assess barriers and deterrents to service and treatment access and utilization.

### Setting

Study procedures took place in 11 Northern Ontario-based communities (see S2 Appendix), including: North Bay, Sudbury, Timmins, Smooth Rock Falls, Kapuskasing, Kenora, Fort Frances, Dryden, Sioux Lookout, Thunder Bay and Sault Ste. Marie. Communities were accessed utilizing the Centre for Addiction and Mental Health (CAMH) Mobile Research Lab (see S3 Appendix). Communities were chosen as they represented a wide range of different situations and characteristics such as size, population demographics, service availability, etc.

### Design

The study utilized a convergent-parallel mixed-methods design [36], employing standardized questionnaires and semi-structured one-on-one interviews and focus groups. We followed a community-based participatory research approach [37–40] where we built and fostered relationships with community members and key informants from service organizations prior to, and maintained these relationships throughout, the data collection phase. Specifically, for each community, we established a main contact/partner/stakeholder (a ‘key informant’) who was involved throughout the entirety of the project. This contact referred other community members and stakeholders from a wide variety of backgrounds and organizations including people with lived/living experience as well as Indigenous scholars and partners. We collaborated with these partners and received ongoing feedback and input on our research design, operational plans, recruitment, etc., as well as elicited recommendations and suggestions for data collection and analysis in each community.

### Eligibility criteria

The study had two separate types of participants who could be eligible (see protocol for detailed criteria [35]): 1) youth, aged 14–25, who have used illicit and/or non-medical prescription drugs for at least 3 months, on at least 10 days in the past month; and 2) key informants who held a formal position in the community where they worked within a service or organization that served, interacted or worked directly with youth who use illicit and/or non-medical drugs. Key informants were comprised of people from a variety of backgrounds and organizations, e.g., nurses and other frontline healthcare staff, outreach workers, peer workers, shelter managers, probation and police officers, Indigenous organization directors, mental health and addiction caseworkers, drop-in center managers, community AA/NA sponsors, members from community drug strategy committees or advisory boards, people with lived and living experience, etc.

### Recruitment

Prior to entering the field, the research team utilized our main key informant within each community to help identify additional health and social service organizations/stakeholders who were willing and able to assist with recruitment once we were in their community. We informed these key informants about the aims and objectives of the study via a standardized email letter. Two researchers visited all organizations (including additional organizations identified in the field) in person to meet key informants and to share our recruitment materials with any newly identified organizations. Following a snow-ball sampling approach, key informants were asked to inform their colleagues, community partners and the youth they work with about the study and provide them with posters and handouts containing study details. Key informants were also asked to assist with organizing focus groups who would meet the research team and undergo data collection procedures at pre-established times. The key informants also assisted the research team with identifying a parking spot in each community for the mobile lab that would be visible and easily accessible, and were asked to participate in a one-on-one interview as a key informant participant.

Beyond utilizing key informants to assist with recruitment, while in each community the study team also directly recruited participants by verbally informing individuals who approached the mobile research lab about the study, and by using a respondent-driven sampling technique where we offered participants an additional incentive to refer eligible peers (see protocol for details [35]).

### Study procedures

Between August and December 2017, two researchers from the study team travelled to each community using the mobile research lab. The lab was parked for approximately one week in each community, depending on participant recruitment success.

Potentially eligible participants were either sent to the mobile lab by key informants who had explained the study to them, were identified by approaching the mobile lab and expressing interest in participating, or were recruited by their peers who had completed data collection procedures.

All potentially eligible participants were screened for eligibility, and underwent a written informed consent process (see protocol for eligibility screener and consent form [35]). Parental consent was not sought for minors (aged 14–16) who participated in the study as they may have been street-involved, disconnected from their parents, or did not want to reveal their drug use to their parents; this approach was line with legal requirements in Ontario, as long as the participants could be considered a mature person, which was approved by CAMH’s Research Ethics Board. After assessing maturity, eligible participants were asked to participate in an anonymous semi-structured, audio-recorded focus group (with three to five participants) or interview (when only one or two participants were eligible at a time). After the focus group/interview session, participants partook in a self-administered questionnaire (see protocol for quantitative and qualitative assessment tools [35]). At the end of the study session, participants were compensated with a $20 gift card and were provided with a list of available addiction services and treatment programs in their community.

The researchers also conducted one-on-one interviews with key informants, either in person or over the phone; Key informants were not provided any compensation.

### Data collection and analysis

In order to maintain confidentiality and anonymity, all participants were provided with a unique participation study code that was utilized to identify them, and all personal identifiers were removed from the data.

Both quantitative and qualitative data were merged, analyzed and interpreted to inform the results. In order to ensure the results were adequately represented and interpreted, an iterative feedback process was undertaken after initial analyses of our results, and key informants were given the opportunity to review the final research findings prior to publication.

#### Quantitative analysis

For the quantitative data obtained from the questionnaire responses, basic descriptive analyses were conducted to characterize the study sample. Using simple frequency and cross-tabulations we examined sociodemographic information, drug use patterns and profiles, and health status/diagnoses. All analyses were conducted using GraphPad Prism (version 8) [41, 42].

#### Qualitative analysis

For the qualitative data, audio recordings were transcribed verbatim and imported into qualitative data management software (NVivo, version 12) [43, 44]. All interview transcripts underwent an inductive thematic analysis whereby key themes were identified and subsequently coded into categories. Initial themes were developed based on our research questions, and discussed and agreed upon as a group. The team developed an original code list based on these themes, and coding was then conducted by two research analysts (CR and MN); additional codes were added based on emergent themes during analyses. When analyzing/comparing the data between the youth and key informants, data saturation was discussed among the two interviewers, and we conducted a crosswalk to ensure a consensus was reached. Codes and themes were included in final analyses when they were introduced or discussed by numerous participants, and from these, select illustrative samples (from both youth and key informants) were chosen to narratively represent the themes.

## Results

Across the 11 Northern Ontario communities visited, there were a total of n = 102 youth, and n = 35 key informants, who completed the qualitative component of our study; n = 100 youth completed the quantitative component.

### Quantitative results

#### Sociodemographic information

Participants ranged in age from 14 to 25, with a mean age of 22.2, and were split relatively equally between males (49%) and females (47%). The vast majority (75%) self-identified their ethnic background as ‘Aboriginal’. Most participants (39%) reported living at their own place. Regarding highest level of education, half (50%) reported they did not (or had yet to) finish high school. As for sources of income in the last month, 61% of participants reported receiving government assistance (welfare and/or disability) as their primary source of income.

#### Drug use patterns and profiles

Besides alcohol, tobacco and cannabis, the most commonly used drugs (reported as ‘use on more than 10 days in the last month’) were any prescription opioids (66%), cocaine (65%), crack-cocaine (52%), benzodiazepines (28%), methamphetamines/crystal meth (24%) and amphetamines/speed/ADHD medication (22%). The majority (67%) of participants indicated that they had injected drugs at least once, while 35% reported ever experiencing an overdose.

Almost all participants reported experiencing ‘problems’ related to their drug use. Specifically, the majority (72%) indicated they had experienced ‘social problems’, followed by ‘financial’ (60%), ‘school-based’ (59%), ‘health’ (45%), ‘work’ (45%), and ‘legal problems’ (42%).

#### Health status

When asked about current health status (reported as ‘in the last 30 days’) utilizing a 5-point Likert scale (i.e., 1 = poor, 2 = fair, 3 = good, 4 = very good, 5 = excellent) the most common category chosen was ‘fair’ for both physical (32%) and mental (30%) health status. Notably, a large proportion (24%) reported ‘poor’ mental health status. In addition, when asked about specific mental health diagnoses, 60% of participants reported ever having received a diagnosis of depression, followed by anxiety (56%), substance use disorder (42%), schizophrenia or psychosis (19%), and a personality disorder (16%); notably, among respondents with a diagnosis of depression, anxiety or substance use disorder (n = 43), 63% reported that they had ever been diagnosed with all three.

### Qualitative results

#### Treatment or service barriers and deterrents

The participants reported a variety of influential factors that affected their desire or ability to utilize available services or treatments in their communities. These factors were primarily categorized into four main themes: Personal; Social; Structural; and Physical.

#### Personal barriers or deterrents

One of the major themes that arose when examining barriers to treatment revolved around personal or individual-level obstacles. For instance, many participants indicated that the main reason they had not sought treatment was because they were unaware of which services were available in their community, did not know how to access them, and that there was generally no information about how to navigate the service system.

Regardless of whether or not participants were aware of what treatments or services were available in their community, many youth participants indicated that they did not attend or utilize them, and expressed apathy towards treatment. Some had not thought about getting help, whereas others communicated that they did not want to get help, were not ‘ready’ to seek treatment, or had a lack of motivation and/or no desire to restrict their drug use. For example, one youth participant responded the following when asked about ever thinking about seeking help for their drug use:

“*Not really*, *because I’m not looking for help right now*. *Because I’m not ready to quit*.*”*

(Youth Participant 44)

Many participants indicated that they wanted to keep using drugs, and that even if they sought treatment or help, or were obliged to go to treatment (e.g., due to court orders, parent’s decisions, etc.), it would be futile. Key informants further suggested that many youth have a hard time admitting that they have a problem or need help, and often believe they can handle their problems on their own, especially when it comes to addiction and/or mental health issues.

“*Nobody really wants to go get help*, *like we have a hard time admitting that we need help*, *especially for things that are like*, *oh*, *whatever*, *I can do it by myself*. *Like you know*, *like mental health or like addictions*, *like nobody wants to admit it*.*”*

(Key Informant 7)

#### Social barriers or deterrents

Another common barrier or deterrent for treatment that arose revolved around social barriers. Both the youth and key informant participants stated that there was a lack of confidentiality and privacy in their communities, and that organizations often shared client information amongst each other. Moreover, many youth indicated that they did not want their friends or family to know that they were seeking treatment. Specifically, fear of being judged/stigmatized, letting the important people in their life down, and social isolation were all articulated reservations. As an example, this was expressed by one key informant when they stated:

“*They [youth] don’t want to acknowledge the family members or repercussions*, *letting their parents down*, *whatever way you want to say*. *Also with their peers or friends they don’t want to be the one that says I've got a problem and then get shunned out*.*”*

(Key Informant 15)

Furthermore, some youth participants and key informants expressed that people are scared of seeking treatment due to the potential consequences of going to the authorities, such as having the police or children’s protective services become involved in their lives. For instance, one youth participant expressed this particular concern:

“…*some of my friends*, *like they have families also*, *and they’re scared to like go forth and make their addictions and problems known to professionals because they’re scared to–you know*, *like Children’s Aid might get called or something like that*.*”*

(Youth Participant 95)

Moreover, many participants noted that their friend groups and/or acquaintances would deter them from seeking help and would sometimes peer-pressure them into skipping appointments or attending treatment. This was specifically the case for those who had to visit treatment centres or services daily (e.g., methadone clinics, pharmacies, needle exchange programs, etc.) as they would often run into acquaintances there. When asked if there was anything that made it difficult for the youth to attend services, one youth participant stated:

“*Friends* …*like if you hang out with a bunch of people who use and there's drugs right in front of you because they're doing it you're going to want to do your drugs and not go*.*”*

(Youth Participant 83)

Some youth reported that they did not want to seek treatment because it would be embarrassing or socially challenging. Specifically, stigma, judgment, discrimination and racism were often reported by the participants as reasons for not seeking or continuing treatment. Perceptions of systemic discrimination and stigmatization of drug use were particularly salient, and especially noted as common among certain service providers, such as hospitals. As such, many participants expressed that service provider attitudes and/or lack of professionalism was a deterrent to using services. For example, one youth participant viewed service providers to be judgmental, and stated:

“*They [service providers] don’t understand as much why people do it*. *They more look at the addiction rather than looking at the reason for the addiction…a lot of my friends don’t want to go and get help because they feel it’s just going to turn into judging*, *especially because if you go to a hospital or something*, *you get found with this kind of drug in your system*, *certain medication they won’t give you even if you need it or anything because even if you’re a past user*, *you might*. *So there’s a lot of stereotype and a lot of criticism*.*”*

(Youth Participant 15)

#### Structural barriers or deterrents

A third key theme that arose when examining barriers or deterrents to treatment regarded structural barriers. Wait lists to get into treatment or to be connected with a service provider were the most commonly reported deterrents. Being put on a wait list directly affected many participants’ motivation to continue to seek treatment. Numerous youth participants expressed that their desire to get help was often fleeting and time-sensitive; if they did not get immediate help when motivated to do so, they would change their minds, forget to attend their appointments, and/or revert back to drug use.

“*The waiting time* … *I know a lot of people bitch about that*. *Like they're in the mood to get clean and then by the time the appointment comes they forget it and then it's done*. *Like they miss the appointment and then they got to do it all over again and then they just say fuck it*, *let's go get high*.*”*

(Youth Participant 76)

Other concerns revolved around a general lack of services available in their small communities, and that existent services are extremely under-resourced. Some places, such as emergency shelters, were often too full and/or had limited capacity to take on new clients. For instance, when asked what services or treatments were available for youth in their community, one key informant stated:

“*The big thing that I always hear is there's not enough*, *not enough beds available at either shelters or with treatment*, *it's just constantly busy*. *And if somebody needs treatment and they wanted to go in like I feel like they should be able to go in right away*.*”*

(Key Informant 26)

Other programs (especially detox/withdrawal management and rehabilitation programs) had limitations on the amount of time a client could stay and use their services; typically, once the client was finished their allotted treatment ‘period’, they were not connected to further service providers.

In addition, limited hours of operation were commonly cited as major barriers to seeking treatment. Most services closed early in the day and/or were only open on certain days of the week. For example, when asked about barriers to accessing services in their community, one youth participant mentioned:

“*The Health Unit is closed on the weekends*, *so that’s become a problem before* …*because that’s two days in a row that it’s closed…and they’re the only place that provide free*, *like*, *supplies*.*”*

(Youth Participant 21)

Other barriers involved administrative or bureaucratic requirements. Not having proper identification was seen as a major barrier to receiving treatment or accessing/retaining necessary social service assistance such as welfare or disability, which was integral to ensuring a steady enough income to secure safe housing and obtain necessities. Some services required membership fees that the youth did not have the money to cover and could not afford. Furthermore, the overall process of seeking help was seen as overly administratively burdensome and difficult to navigate which would directly deter participants from trying. Other respondents indicated that even if they knew where to go or who to speak to, there was just not enough help and no specific go-to person that could assist them. For example, one youth participant expressed this:

“*Ontario Works [OW] sure*, *like welfare is good and all*, *but it’s extremely hard to get on it if you’re homeless and you’re not in school*. *As a youth*, *I tried to get on OW after I lost my job*, *after I had moved out of [service name] that last time*, *and I tried three times and they wouldn’t let me on even though I brought my eviction papers to them*, *I brought my ROE [record of employment] to them*. *I told them exactly what was going on*, *I told them exactly why I wasn’t going to school*. *They gave me a list of things to do to qualify for OW*, *I got all of them done*. *They gave me 2 days*. *I still managed to get every single one of them done in those 2 days and they still didn’t let me on*.*”*

(Youth Participant 5)

#### Physical barriers or deterrents

The final overarching theme that arose when examining barriers or deterrents to treatment focused on physical barriers. In particular, a common concern regarded geographical isolation, as well as a lack of transportation. Many youth participants reported not owning a vehicle and relying on public transportation to get around the city, however, buses ran infrequently, and participants did not have money to afford bus fare so they could not make their appointments. For instance, when asked if there were any problems trying to access services in their community, one youth participant stated:

“*Usually transportation if I lived too far from it is always the main concern…I take the bus because I can’t drive and I don’t always have bus fare or money to buy a monthly bus pass*.*”*

(Youth Participant 4)

Moreover, many youth and key informant participants indicated that due to a lack of services in their home community, the youth often had to travel to other communities to access services. This was viewed as especially difficult due to lack of transportation and the remoteness of certain communities–particularly Northern First Nations reserves–which were difficult to reach. For instance, when asked if their geographic location affected their ability to access services, one youth participant stated:

“*For me*, *it’s pretty much an isolated reserve*, *so that’s pretty hard*. *Two times harder than it was in Timmins for me*. *It’s a fly-to area*.*”*

(Youth Participant 23)

Many youth participants expressed that they had travelled to certain communities to get help (or had been brought there by nature of being involved in the criminal justice system), but once they had finished treatment (or were released from jail), were not able to travel back to the remote communities they came from. This contributed to participants being transient and/or homeless.

In addition, a lack of mobility overall, as well as weather concerns and a lack of weather-appropriate clothing were common issues reported by both youth and key informant participants as major barriers. For example, when asked whether they had personally experienced problems accessing services one youth participant indicated:

“*During the summer no*, *but during the winter it can be a big problem like I’m not going to walk 25 minutes to a service in blistering cold weather when I don’t own a winter jacket or winter boots*.*”*

(Youth Participant 18)

#### Treatment or service needs and wants

The youth participants in each community expressed a number of desires for treatments or services that they would need or want to have available. These needs included references for scaling-up services provided by existing organizations, as well as requests for the implementation of specialized and/or new programs and services; these desires were categorized into: harm reduction-based programs; low-threshold programs; specialized and/or group-specific programs; and counselling and/or peer-based programs.

#### Harm reduction-based programs

The most common request made by both youth and key informant participants when asked what treatment or services they would like to see in their community focused on harm reduction-based programming. For example, many participants suggested a need for a safe injection site or somewhere similar where they could go consume their drugs as well as get their drugs checked or tested to ensure that they were safe.

“*I strongly suggest a safe injection site because we see*, *on a daily basis*, *we see needles on a couple of streets and everything now and I wouldn't want my kids stepping on it*.*”*

(Youth Participant 59)

Scaling-up of needle exchange programs and naloxone provision were also articulated needs, particularly because youth did not have the money to afford buying clean needles from pharmacies. Some participants expressed a need for more places to dispose of their used needles since there were only a few places that had bins to do so in their community, and that this consequently led them to littering their used needles on the ground. Mobile outreach harm-reduction based services which deliver clean needles, necessary health supplies and wound care, as well as dispose of used paraphernalia, were proposed as services that would be particularly helpful. For example, one youth participant suggested:

“*Have more outreach vehicles*. *Even they can go around to the using houses*, *and collect the dirty ones [needles]*, *and get them clean*, *and keep it sterile*.*”*

(Youth Participant 1)

Lastly, some participants requested general knowledge on safer use practices.

“*Somebody that can…like a service that shows you how to… use drugs safely*, *like harm reduction*.*”*

(Youth Participant 78)

#### Low-threshold programs

The second most common theme that emerged regarding service and treatment needs in the communities referred to low-threshold programs (i.e., programs that do not impose abstinence from drug use and remove bureaucratic or administrative barriers to access). To this end, the most common recommendation by youth and key informants was a youth-specific drop-in centre that was easily accessible. For instance, when asked if there were any services in their community that they would want to have, one youth participant stated:

“*Well*, *first of all*, *they don’t even have drop-in centres*. *They don’t have drop-in centres over here*, *like somewhere you can go and chill out and not have to pay money*.*”*

(Youth Participant 26)

Some of the youth participants recognized that there was not a lot to do in their small towns, so they would use drugs as a form of entertainment. As such, other suggestions included a need for increased community-based and/or organized activities. As an example, when asked what would be the most helpful for youth in the community, one participant suggested:

“*Maybe like more activities*, *like sports activities*, *like free–like some kind of organized activities more often*. *Like there’s nothing around here*.*”*

(Youth Participant 25)

In addition, flexibility and increased hours of operation–specifically 24-hour availability–were commonly suggested. Many youth and key informant participants noted that services are only open limited hours which are not conducive to their lifestyle. One youth participant, like many others, made this need clear when they indicated:

“*I think that they should have a 24-hour clinic for people*, *you know*, *just in case someone needs a naloxone kit*, *or just in case someone decides they’re going to use a dirty needle*, *and then they get hepatitis or AIDS*. *Like they don’t know*, *it could save someone’s life…like I think that if they had a more flexible sort of an organization that could actually be there when people really actually need it*, *instead of when it’s convenient for them*.*”*

(Youth Participant 43)

Importantly, a place that offers access to treatment as well as wrap-around services for health and drug use-related issues, in one centralized location, was seen as something that would be most beneficial for youth. This ‘centralized’ location was seen as crucial to alleviate the burden of needing to travel to different services and in order to mitigate geographical restrictions. One key informant reiterated the need for such a comprehensive and consolidated service:

“*Well*, *the walk-in centre like we were talking about…they need more of those…also provide like a safe injection site*, *all that under one roof*. *One of the problems*, *like I said*, *sending people around to different roofs is a bad idea*. *You need to keep everything in one place*.*”*

(Key Informant 27)

#### Specialized and/or group-specific programs

Another common theme that emerged when examining treatment or service needs and wants within the communities revolved around specialized programming. For instance, several youth participants expressed a need for more tailored and youth-oriented programs. Many of the available programs in the communities were either targeted specifically towards adults (some of which would not accept youth), or accepted people from all ages and provided generalized services (which were thus seen as irrelevant or unhelpful for youth). For instance, when asked if there were any services they needed in their community, one youth participant stated:

“*And we should have a shelter just for young*, *not mixed with the adults*. *We should have a shelter here for just the young homeless people like the way they have it in Toronto*. *They have homeless shelters for the younger ones and the older ones because when the young ones come here they're learning off the older people and that's how they pick up needles and stuff*.*”*

(Youth Participant 74)

Some of the participants who self-identified as Indigenous suggested that more cultural or traditional-based programming would be beneficial for them as it would allow them to get in touch with their roots and address their issues using the teachings of their culture. Other specific needs included having a safe, open and non-discriminatory space for those who identify as Lesbian, Gay, Bisexual, Transgender, or Queer (LGBTQ) to go and seek treatment that is relevant to the specific issues they were facing. For instance, when asked what services they would want to see in their community, one participant stated:

“*There should be a place for people that are gay*, *bisexual*, *whatever*, *the LGBT community and stuff like that*. *Just a separate place for them to go and feel comfortable and not afraid too*.*”*

(Youth Participant 10)

In addition, cut-off ages where certain programs cannot accept clients under a certain age, or are mandated to remove people from the program once they become a certain age, were seen as unaccommodating. As such, some participants suggested eliminating cut-off requirements and expressed a need for an increase in transitional-based programming to address this issue. Many key informants also re-iterated the need for youth-specific and transitional programming. As an example, one key informant commented on the struggles of helping youth where services are predominately adult-based:

“*We need … mental health services and addiction services targeted specific to youth…I'd love to have counsellors that are trained in counselling youth…we have no pediatric mental health counsellors…I think it really is being in a smaller northern community we don't have the same youth services and it is*, *it's all about having targeted to youth*. *I hate having to try and access adult care–whether it's to do with mental health*, *addictions*, *LGBT issues–I’m trying to navigate the adult system for kids which I hate*. *It's not the same*, *they're not the same people*, *they need someone who knows kind of the teenage brain and some of the things they're dealing with*.*”*

(Key Informant 19)

Gender-specific programs were also suggested. This included maternal or pregnancy-based–programs that address women’s specific needs in a safe and effective manner. For instance, when asked if there was anything they would like to change or improve about the services in their community, one youth participant revealed:

“*They need more programs…that support pregnant woman and this situation [being addicted to drugs while pregnant]*. *There should be much more of this kind of programming…it's so sad*.*”*

(Youth Participant 92)

Family-based treatment and services were further suggested as some participants had tumultuous family and home lives, and/or would engage in substance use and criminal activities with their family members. Further, some participants expressed that there were no treatments or services that were specifically tailored to addressing their drug of choice such as cocaine, speed, or crystal meth, and that a program that was drug-specific would be helpful.

“*We need something to help everyone get off this crystal meth*. *There’s nothing to help people get off of crystal meth*. *There’s nothing*, *man*. *You have to do it cold turkey*.*”*

(Youth Participant 36)

#### Counselling and/or peer-based programs

Further needs that arose focused on a desire to have more counselling-based services. Many youth participants articulated that they just wanted “someone to talk to”, and someone who was willing to listen to them about the problems they were experiencing. For instance, when asked if there was anything else they needed that they did not have access to in terms of services for their drug use, one youth participant stated:

“*Probably counselling*. *Somebody to talk to*. *Somebody who wants to talk to us*.*”*

(Youth Participant 29)

In addition, it was noted that services would be more helpful if they were non-discriminatory, and run by people with lived experience or their peers so that the youth would feel more comfortable opening up and relating to someone who has gone through what they have. As an example, when asked what service would be the most beneficial, one key informant mentioned the importance of using peer workers:

“*Peer workers*. *So have a youth as the outreach worker cause who knows a youth better than a youth*.*”*

(Key Informant 11)

This was also seen as a way for the youth to have their voices heard since they often felt as though they were not included in the decisions that affect them.

## Discussion

The purpose of this study was to examine the barriers, gaps and needs related to treatment and service provision for youth who use illicit and non-medical prescription drugs in Northern Ontario communities. The goal was to solicit valuable input from youth, as well as key informants who work with youth, on how to improve the service system to meet the needs of youth. In addition, we utilized a community-based participatory research approach with an emphasis on connecting with community members, viewing them as equal partners in the research and embedding the research in the specific context of each community; this approach has been found effective, especially when researching marginalized populations [39, 40, 45].

Findings from this study corroborate many of the conclusions found in existing literature on this topic. For example, remote communities have been found to have critical service gaps for addictions and mental health needs [25, 26, 28, 46], and Northern and rural communities often have few mental health and addictions services; where these do exist, they are generally fragmented and disconnected from one another [47, 48]. Moreover, an overall lack of services specific to youth addicted to drugs, and a need for prevention, early intervention activities and low-threshold/easily accessible substance use treatments that are youth-oriented has been identified [15, 18, 49, 50]. Other studies suggest that there are common barriers that impact youth and directly affect their decisions, specifically when it comes to seeking treatment such as lack of awareness or motivation, stigma, wait times, paper work, etc. [15, 49, 51].

Although each community is unique and presents their own individual needs, overall, many of the outcomes, sentiments and suggestions were similar across all communities. Specifically, general apathy towards confronting their drug use was common among youth; yet this is not a novel finding since youth have been found less likely to seek help specifically for mental health or substance-use-related issues compared to their older counterparts, and tend to be self-reliant when it comes to dealing with these problems [52, 53]. However, studies have found that among youth, perceived benefits of treatment can predict help-seeking behavior and can outweigh perceived barriers [54]. As such, increasing motivation to seek treatment among youth, and promoting and reinforcing the benefits of treatment in a way that is most likely to resonate with them is necessary in order to increase its appeal and uptake, and should be a focus when it comes to treating youth with substance use problems [55–58]. In order to achieve this, it is imperative to have open discussions with youth about the benefits of seeking treatment and speaking to someone about their problems, especially since adolescence is a critical time point for physical and psychological development, and is a high-risk period for drug use initiation [2, 3, 59, 60].

Nonetheless, even when youth become motivated to seek treatment and understand its benefits, stigmatization remains a major deterrent. Fear of stigmatization and beliefs about the lack of helpfulness of treatments and services for addiction and mental health issues has been correlated with help-seeking intentions among youth [52, 53], and many studies have reported this as a specific barrier for treatment [15, 50, 51, 61]. Drug use is inherently stigmatized, and particularly so among youth; studies have shown that stigmatization experienced in youth can carry into adulthood and can affect personal drug use experiences and trajectories [61–63]. As such, it is important to tackle stigmatization and offer services that youth perceive as socially inclusive and approachable. One way to facilitate this is to offer services run by non-discriminatory and empathetic counsellors and/or peers, and to recognize that lived experience provides unique contributions towards positive change and ultimately, the reduction of stigma [64, 65].

When it comes to treatment and service needs, low-threshold and/or harm reduction-based services were the most commonly reported need. Low-threshold programs have been defined differently [66], but essentially include ‘inviting’ atmospheres, effective client engagement, confidential service delivery, tailored services, and peer support to reduce stated barriers to service access. As a primary example, many youth participants suggested drop-in centers with on-the-spot help available 24 hours a day, run by peer counsellors as a service they would use and benefit from. Notably, there have been a number of low-threshold harm reduction-based interventions implemented in some of these communities since our data was collected. For example, supervised consumption and/or overdose prevention sites (both legally sanctioned and non-legally sanctioned) as well as rapid access addiction medicine clinics (i.e., low-barrier, walk-in clinics where patients can go to get help for a substance use disorder without referrals) have been executed in a number of Northern Ontario communities such as Thunder Bay and Sudbury [67–72].

Moreover, there is an overall low-barrier ‘integrated youth services’ movement unfolding in Ontario (and Canada more broadly), which includes a number of initiatives and programs that provide easy access to brief interventions, peer support, care navigation, primary care and a point of access to higher intensity services for both mental health and addictions issues for youth [64, 73–76]. For example, in Ontario specifically, ‘youth wellness hubs’ have now been implemented in a number of communities across Ontario (including in the North), which were established to serve as integrated ‘one-stop-shops’ for youth aged 12–25 who are dealing with mental health and addictions issues; these include many of the services that youth suggested would be beneficial to them (such as peer services, outreach, system navigation, etc.) [64, 73]. Therefore, since our data collection concluded, there have been a number of youth-oriented interventions that have been implemented which are slowly expanding and ideally addressing many of the issues that youth reported experiencing.

Although these are positive efforts, many have faced strong political opposition which has hindered their effectiveness, and they have not been implemented equally across all communities (e.g., there are currently only 10 ‘youth wellness hubs’ across all of Ontario). As such, more needs to be done to address the youths’ articulated needs.

Additional research on this important topic is highly needed. Studies that geographically and/or spatially map all treatment and service providers available in Northern Ontario would be helpful to gaining a better understanding of the availability of these services. Moreover, quantitative studies examining treatment utilization and transition of care within each community using publically-available health record data and databases (e.g., Ontario Health Insurance Plan (OHIP); Institute for Clinical Evaluative Sciences (ICES), etc.) could help better inform whether or not and how often youth are seeking help for drug use in their communities. Of note, involving people with lived and living experience, as well as Indigenous people and culturally-specific organizations, throughout the entirety of such studies (including in conceptualization, design, implementation and analysis) is crucial to ensuring that the results truly reflect the experiences of the participants and that the knowledge created is accessible to those that can benefit from it.

The results of this study reflect the perceived experiences of the participants in their unique community-based settings, and were affirmed by key informants during the feedback process. However, the results should be interpreted with caution and limitations to this research must be noted. This study was a mixed-methods study, and as such, is susceptible to biases inherent in self-report data such as memory or recall bias, response bias, interpretation of the questions, and social desirability; this may be particularly true since focus groups have a tendency to elicit a dominant voice, and the potential for posturing for the microphone exists. Further, some participants may have lied about their age due to the incentive of the participant honorarium, but measures to mitigate this possibility were taken. Moreover, given that drug use is heavily stigmatized, particularly in smaller communities, participants may have under-reported dimensions of their drug use and any discrimination or prejudice experienced. The information gleaned from this study is also not generalizable for a number of reasons: the study only reports on the 11 Northern Ontario communities visited, so the findings are specific to youth who reside in these communities; the number of participants recruited from each community widely varied due to a number of factors, e.g., lack of eligible participants, weather conditions, level of engagement with community partners, number of services available in the community, etc.; the sample was purposeful, and many of the youth had pre-existing relationships with the service providers in the community and may therefore under-represent those who have no connection to the service system, although we utilized a respondent-driven sampling technique in order to mitigate this bias. Moreover, the mobile lab was parked in pre-determined places where it would be the most visible to our target population, and potential participants may have felt insecure or uncomfortable to be seen in the mobile lab. Further, we may not have reached potential participants who live in more remote areas and cannot travel or reach the mobile lab due to a lack of transportation, which was pinpointed as a major barrier to accessing services.

## Conclusion

The findings from this study suggest that youth who use illicit and non-medical prescription drugs in Northern Ontario communities would benefit from community-based implementation of low-threshold services such as a drop-in centers/emergency shelters tailored specifically to youth–available and open around the clock–which provide education about the benefits of treatment as well as service system navigation, activities, and wrap-around services. These services should mandatorily provide rapid-access medical assessment/treatment, counselling and programs run by peers and non-judgmental and empathetic professionals in order to be optimally effective for youth. In addition, it would benefit both youth as well as community service providers to implement a system or arrangement (e.g., monthly community consultations) that includes youth in the decision making process and which improves integration and collaboration across organizations and sectors within each community. This would allow for a deeper understanding of the ongoing issues and needs of youth in each community, and provide for better community connections to address these needs.

This study helps to better understand the needs of the youth who reside in Northern Ontario communities, which is essential since it was conveyed that many of the currently available services are not relevant or effective for them. Moreover, although our findings may not be generalizable, the knowledge gained is relevant and can be applied to many remote and rural community settings across Canada (and North America more broadly). Thus, the presented results have the potential to aid in evidence-based decision making for the development and implementation of future services and interventions for youth who use illicit drugs which actually reach the youth, address their articulated needs, and help them deal with their substance use issues.

## Supporting information

S1 Appendix Map of 14 Local Health Integration Networks (LHINs) across Ontario. (DOCX)

S2 Appendix Map of communities visited across Northern Ontario. (DOCX)

S3 Appendix Centre for Addiction and Mental Health (CAMH) mobile research lab. (DOCX)

## References

[1] United Nations Office on Drugs and Crime (UNODC). World Drug Report 2018. Vienna, Austria: United Nations Office on Drugs and Crime (UNODC); 2019.

[2] Hall WD, Patton G, Stockings E, Weier M, Lynskey M, Morley KI, et al Why young people's substance use matters for global health. The Lancet Psychiatry 2016, 3(3):265–279. 10.1016/S2215-0366(16)00013-4 26905482

[3] Erskine HE, Moffitt TE, Copeland WE, Costello EJ, Ferrari AJ, Patton G, et al A heavy burden on young minds: the global burden of mental and substance use disorders in children and youth. Psychol Med 2014, 45(7):1551–1563. 10.1017/S0033291714002888 25534496 PMC5922255

[4] Health Canada. Canadian Tobacco, Alcohol and Drugs Survey (CTADS): Summary of results for 2017. Ottawa, ON: Health Canada; 2019.

[5] Canadian Centre for Substance Abuse (CCSA). A Drug Prevention Strategy for Canada's Youth. Ottawa, ON: Canadian Centre for Substance Abuse (CCSA); 2007.

[6] Canadian Centre on Substance Abuse (CCSA). Substance Abuse in Canada: Youth in Focus. Ottawa, ON: Canadian Centre on Substance Abuse (CCSA); 2007.

[7] Canadian Centre on Substance Abuse (CCSA). Cross-Canada Report on Student Alcohol and Drug Use: Technical Report Ottawa, ON: Canadian Centre for Substane Abuse (CCSA); 2011.

[8] Boak A, Hamilton HA, Adlaf EM, Mann RE. Drug Use Among Ontario Students, 1977–2017: Detailed findings from the Ontario Student Drug Use and Health Survey (OSDUHS) *CAMH Research Document Series*. Toronto, ON: Centre for Addiction and Mental Health (CAMH); 2017.

[9] Ialomiteanu AR, Hamilton HA, Adlaf E, M., Mann RE. CAMH Monitor E-Report: Substance Use, Mental Health and Well-Being Among Ontario Adults, 1977–2017. Toronto, ON: Centre for Addiction and Mental Health (CAMH); 2018.

[10] Gomes T, Khuu W, Martins D, Tadrous M, Mamdani MM, Paterson JM, et al Contributions of prescribed and non-prescribed opioids to opioid related deaths: population based cohort study in Ontario, Canada. BMJ 2018, 362:k3207 10.1136/bmj.k3207 30158106 PMC6113771

[11] Russell C, Firestone M, Kelly L, Mushquash C, Fischer B. Prescription opioid prescribing, use/misuse, harms and treatment among Aboriginal people in Canada: a narrative review of available data and indicators. Rural Remote Health 2016, 16(4). 27871180

[12] Rush B, Kirkby C, Furlong A. North East Local Health Integration Network Addiction Services Review. North Bay, ON: North East Local Health Integration Network 2016.

[13] Kurdyak P, Binu J, Zaheer J, Fischer B. Patterns of Methadone Maintenance Treatment Provision in Ontario: Policy Success or Pendulum Excess?. Canadian Family Physician 2018, 64(2):95–103. 29449263 PMC5964406

[14] Rowan M, Poole N, Shea B, Gone JP, Mykota D, Farag M, et al Cultural interventions to treat addictions in Indigenous populations: findings from a scoping study. Substance Abuse Treatment, Prevention, and Policy 2014, 9(1):34. 10.1186/1747-597X-9-34 PMC4158387 25179797

[15] Brownlie EB, Chaim G, Heffernan O, Herzog T, Henderson J. Youth Services System Review: Moving From Knowledge Gathering to Implementation Through Collaboration, Youth Engagement, and Exploring Local Community Needs. Can J Commun Ment Health 2017, 36(2):133–149.

[16] Mental Health and Addictions Leadership Advisory Council. Better Mental Health Means Better Health: Annual Report of Ontario’s Mental Health & Addictions Leadership Advisory Council Toronto, ON: Mental Health and Addictions Leadership Advisory Council,; 2015.

[17] Winters KC, Botzet AM, Fahnhorst T. Advances in Adolescent Substance Abuse Treatment. Curr Psychiatry Rep 2011, 13(5):416–421. 10.1007/s11920-011-0214-2 21701838 PMC3166985

[18] Chiefs of Ontario. Ontario Region first nations Addiction Service Needs Assessment: Final Report. Ottawa, Ontario: First Nations and Inuit Health Branch of Health Canada & Chiefs of Ontario; 2009.

[19] North East Local Health Integration Network (LHIN). 2016/2017 Annual Report. North Bay, ON: North East Local Health Integration Network; 2018.

[20] North West Local Health Integration Network (LHIN). 2015–2016 North West Local Health Integration Network (LHIN) Annual Business Plan Thunder Bay, ON: North West Local Health Integration Network (LHIN); 2017.

[21] Luce J, Strike C. A Cross-Canada Scan of Methadone Maintenance Treatment Policy Developments. Toronto, ON: Canadian Executive Council on Addictions; 2011.

[22] Anderson TJ, Saman DM, Lipsky MS, Lutfiyya MN. A cross-sectional study on health differences between rural and non-rural U.S. counties using the County Health Rankings. BMC Health Serv Res 2015, 15(1):441. 26423746 10.1186/s12913-015-1053-3 PMC4590732

[23] Strasser R. Rural health around the world: challenges and solutions. Fam Pract 2003, 20(4):457–463. 10.1093/fampra/cmg422 12876121

[24] Weaver KE, Geiger AM, Lu Lingyi, Case LD. Rural-urban disparities in health status among US cancer survivors. Cancer 2013, 119(5):1050–1057. 10.1002/cncr.27840 23096263 PMC3679645

[25] Sibley LM, Weiner JP. An evaluation of access to health care services along the rural-urban continuum in Canada. BMC Health Serv Res 2011, 11(1):20. 21281470 10.1186/1472-6963-11-20 PMC3045284

[26] Ryan-Nicholls KD, Haggarty JM. Collaborative mental health care in rural and isolated Canada: Stakeholder feedback. J Psychosoc Nurs Ment Health Serv 2007, 45(12):37–45. 18246862 10.3928/02793695-20071201-08

[27] Calloway M, Fried B, Johnsen M, Morrissey J. Characterization of rural mental health service systems. The Journal of Rural Health 1999, 15(3):296–307. 10.1111/j.1748-0361.1999.tb00751.x 11942562

[28] Rost K, Fortney J, Fischer E, Smith J. Use, quality, and outcomes of care for mental health: The rural perspective. Med Care Res Rev 2002, 59(3):231–265. 10.1177/1077558702059003001 12205828

[29] Borders TF, Booth BM, Stewart KE, Cheney AM, Curran GM. Rural/Urban Residence, Access, and Perceived Need for Treatment Among African American Cocaine Users. The Journal of Rural Health 2015, 31(1):98–107. 10.1111/jrh.12092 25213603 PMC4280311

[30] Hsia R, Shen Y-C. Possible Geographical Barriers to Trauma Center Access for Vulnerable Patients in the United States: An Analysis of Urban and Rural CommunitiesTrauma Center Access for Vulnerable Patients. JAMA Surgery 2011, 146(1):46–52. 10.1001/archsurg.2010.299 PMC3121679 21242445

[31] Smalley KB, Yancey CT, Warren JC, Naufel K, Ryan R, Pugh JL. Rural mental health and psychological treatment: A review for practitioners. Journal of clinical psychology 2010, 66(5):479–489. 10.1002/jclp.20688 20222125

[32] Regan S, Wong ST. Patient perspectives on primary health care in rural communities: effects of geography on access, continuity and efficiency. Rural Remote Health 2009, 9(1):1142 19298094

[33] Amartey A, Chium M, Gatov EJ, Guttmann A, Lebenbaum M, Kurdyak P, et al The Mental Health of Children and Youth in Ontario: 2017 Scorecard. Toronto, ON: Mental Health and Addictions Scorecard and Evaluation Framework (MHASEF), Institute of Clinical Evaluative Sciences (ICES); 2017.

[34] Statistics Canada. Census Profile, 2016 Census: Ontario Ottawa, ON: Statistics Canada; 2016.

[35] Varatharajan T, Sabioni P, Russell C, Henderson J, Fischer B, Miles S, et al Assessing service and treatment needs of young people who use illicit and non-medical prescription drugs living in Northern Ontario, Canada [version 1; peer review: 1 approved with reservations]. F1000Research 2018, 7(1644). 10.12688/f1000research.16464.2 PMC7025771 32117563

[36] Creswell JW, Creswell JD: Research design: Qualitative, quantitative, and mixed methods approaches: Sage publications; 2017.

[37] Satcher D: Methods in community-based participatory research for health: John Wiley & Sons; 2005.

[38] Wallerstein NB, Duran B. Using community-based participatory research to address health disparities. Health Promot Pract 2006, 7(3):312–323. 10.1177/1524839906289376 16760238

[39] Israel BA, Schulz AJ, Coombe CM, Parker EA, Reyes AG, Rowe Z, et al Community-based participatory research. Urban Health 2019:272.

[40] Israel BA, Coombe CM, Cheezum RR, Schulz AJ, McGranaghan RJ, Lichtenstein R, et al Community-Based Participatory Research: A Capacity-Building Approach for Policy Advocacy Aimed at Eliminating Health Disparities. Am J Public Health 2010, 100(11):2094–2102. 10.2105/AJPH.2009.170506 20864728 PMC2951933

[41] Motulsky H: Analyzing data with GraphPad prism: GraphPad Software Incorporated; 1999.

[42] GraphPad Prism. 7.02 GraphPad Software. 2017.

[43] Gibbs GR: Qualitative Data Analysis: Explorations with NVivo. Buckingham: Open University; 2002.

[44] QSR International Pty Ltd. NVivo qualitative data analysis software: Version 12. 2018.

[45] Holkup PA, Tripp-Reimer T, Salois EM, Weinert C. Community-based participatory research: an approach to intervention research with a Native American community. ANS Adv Nurs Sci 2004, 27(3):162–175. 10.1097/00012272-200407000-00002 15455579 PMC2774214

[46] Public Health Ontario. Open Minds, Healthy Minds: Ontario’s Comprehensive Mental Health and Addictions Strategy. Toronto, ON: Public Health Ontario; 2011.

[47] Durbin J, Lin E, Zaslavska N. Impact Study, Final Report: A study of hospital emergency service use, crisis service delivery and police response after mental health system enhancements Toronto, Ontario, Canada: Health Systems Research and Consulting Unit, Centre for Addiction and Mental Health; 2010.

[48] Brundisini F, Giacomini M, DeJean D, Vanstone M, Winsor S, Smith A. Chronic disease patients' experiences with accessing health care in rural and remote areas: a systematic review and qualitative meta-synthesis. Ontario health technology assessment series 2013, 13(15):1–33. 24228078 PMC3817950

[49] Chaim G, Henderson JL, Brownlie EB. Youth services system review. Toronto, ON; 2013.

[50] Chaim G, Henderson J. Youth System Services Review Phase 2 Report: A review of the continuum of Ontario services addressing substance use available to youth age 12–24. Toronto, ON; 2014.

[51] Wisdom JP, Cavaleri M, Gogel L, Nacht M. Barriers and facilitators to adolescent drug treatment: Youth, family, and staff reports. Addict Res Theory 2011, 19(2):179–188.

[52] Gulliver A, Griffiths KM, Christensen H. Perceived barriers and facilitators to mental health help-seeking in young people: a systematic review. BMC Psychiatry 2010, 10(1):113. 21192795 10.1186/1471-244X-10-113 PMC3022639

[53] Yap MBH, Wright A, Jorm AF. The influence of stigma on young people’s help-seeking intentions and beliefs about the helpfulness of various sources of help. Soc Psychiatry Psychiatr Epidemiol 2011, 46(12):1257–1265. 10.1007/s00127-010-0300-5 20938638

[54] O'Connor PJ, Martin B, Weeks CS, Ong L. Factors that influence young people's mental health help-seeking behaviour: a study based on the Health Belief Model. J Adv Nurs 2014, 70(11):2577–2587. 10.1111/jan.12423 24720449

[55] Peterson PL, Baer JS, Wells EA, Ginzler JA, Garrett SB. Short-term effects of a brief motivational intervention to reduce alcohol and drug risk among homeless adolescents. Psychology of Addictive Behaviors 2006, 20(3):254–264. 10.1037/0893-164X.20.3.254 16938063

[56] Schroder R, Sellman D, Frampton C, Deering D. Youth retention: Factors associated with treatment drop-out from youth alcohol and other drug treatment. Drug and Alcohol Review 2009, 28(6):663–668. 10.1111/j.1465-3362.2009.00076.x 19930021

[57] Battjes RJ, Gordon MS, O'Grady KE, Kinlock TW, Carswell MA. Factors that predict adolescent motivation for substance abuse treatment. Journal of Substance Abuse Treatment 2003, 24(3):221–232. 10.1016/s0740-5472(03)00022-9 12810143

[58] DiClemente CC, Schlundt D, Gemmell L. Readiness and Stages of Change in Addiction Treatment. The American Journal on Addictions 2004, 13(2):103–119. 10.1080/10550490490435777 15204662

[59] Degenhardt L, Stockings E, Patton G, Hall WD, Lynskey M. The increasing global health priority of substance use in young people. The Lancet Psychiatry 2016, 3(3):251–264. 10.1016/S2215-0366(15)00508-8 26905480

[60] Perlmutter AS, Bauman M, Mantha S, Segura LE, Ghandour L, Martins SS. Nonmedical prescription drug use among adolescents: global epidemiological evidence for prevention, assessment, diagnosis, and treatment. Curr Addict Rep 2018:1–8. 10.1007/s40429-018-0194-y PMC6135108 30221120

[61] Adlaf EM, Hamilton HA, Wu F, Noh S. Adolescent stigma towards drug addiction: Effects of age and drug use behaviour. Addict Behav 2009, 34(4):360–364. 10.1016/j.addbeh.2008.11.012 19097707

[62] Corrigan P. How stigma interferes with mental health care. Am Psychol 2004, 59(7):614–625. 10.1037/0003-066X.59.7.614 15491256

[63] Gale F: Tackling the stigma of mental health in vulnerable children and young people In: *Mental health interventions and services for vulnerable children and young people*. edn. Edited by Vostanis P. London, UK: Jessica Kingsley Publishers; 2007: 58–80.

[64] Halsall T, Manion I, Iyer SN, Mathias S, Purcell R, Henderson J: Trends in mental health system transformation: Integrating youth services within the Canadian context In: *Healthcare management forum*: *2019*: SAGE Publications Sage CA: Los Angeles, CA; 2019: 51–55. 10.1177/0840470418808815 30799661

[65] Barton J, Henderson J. Peer support and youth recovery: a brief review of the theoretical underpinnings and evidence. Canadian Journal of Family and Youth/Le Journal Canadien de Famille et de la Jeunesse 2016, 8(1):1–17.

[66] Mofizul Islam M, Topp L, Conigrave KM, Day CA. Defining a service for people who use drugs as ‘low-threshold’: What should be the criteria? Int J Drug Policy 2013, 24(3):220–222. 10.1016/j.drugpo.2013.03.005 23567101

[67] Mitra S, Rachlis B, Krysowaty B, Marshall Z, Olsen C, Rourke S, et al Potential use of supervised injection services among people who inject drugs in a remote and mid-size Canadian setting. BMC Public Health 2019, 19(1):284 10.1186/s12889-019-6606-7 30849946 PMC6408761

[68] Sawula E, Greenaway J, Olsen C, Jaun A, Flanagan Q, Leiterman A, et al Opioid Use and Impacts in Thunder Bay District. Thunder Bay, ON: Thunder Bay District Health Unit; 2018.

[69] Kerr T, Mitra S, Krysowaty B, Marshall Z, Olsen C, Rachlis B, et al Ontario Integrated Supervised Injection Services Feasibility Study (OISIS). Thunder Bay, ON: Thunder Bay Drug Strategy 2017.

[70] Berryman A. The Merits of Supervised Injection Facilities: A Case for Sudbury and Northern Ontario Thunder Bay, ON: Northern Policy Institute 2016.

[71] Diaczuk D. Overdose Prevention Site now open 2018. TB Newswatch Available from https://www.tbnewswatch.com/local-news/overdose-prevention-site-now-open-4-photos-1138000. Cited 17 June 2019

[72] Rutherford K. Underground supervised injection site in Sudbury pops up in Louis st. neighborhood 2019. CBC News Available from https://www.cbc.ca/news/canada/sudbury/stigma-opioid-injection-underground-supervised-1.5158321. Cited 17 June 2019.

[73] Youth Wellness Hubs Ontario. Youth Wellness Hubs Ontario: A Primer. Toronto, ON: Youth Wellness Hubs Ontario 2017.

[74] YouthCan IMPACT. YouthCan IMPACT. 2017. Available from http://www.youthcanimpact.com/research-project. Cited 02 July 2019.

[75] Foundry BC. Foundry, B.C. 2019. Available from https://foundrybc.ca/who-we-are/. Cited 02 July 2019.

[76] ACCESS Open Minds. ACCESS Open Minds. 2019. Available from http://accessopenminds.ca/about/what-is-access/. Cited 02 July 2019.

