# Supplementary figures and images for: Assessing service and treatment needs and barriers of youth who use illicit and non-medical prescription drugs in Northern Ontario, Canada
Source: PLoS One. 2019 Dec 5;14(12):e0225548. doi: 10.1371/journal.pone.0225548 (PMC6894813; doi:10.1371/journal.pone.0225548)

## S1 Appendix

### Map of 14 Local Health Integration Networks (LHINs) Across Ontario


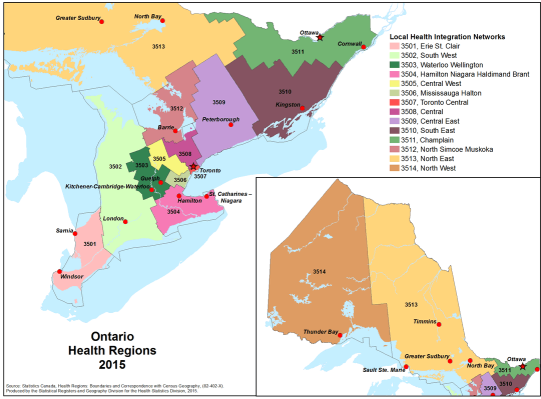

Supplement: S1 Appendix — (DOCX) [file pone.0225548.s001.docx]

## S2 Appendix

### Map of Communities Visited Across Northern Ontario

##
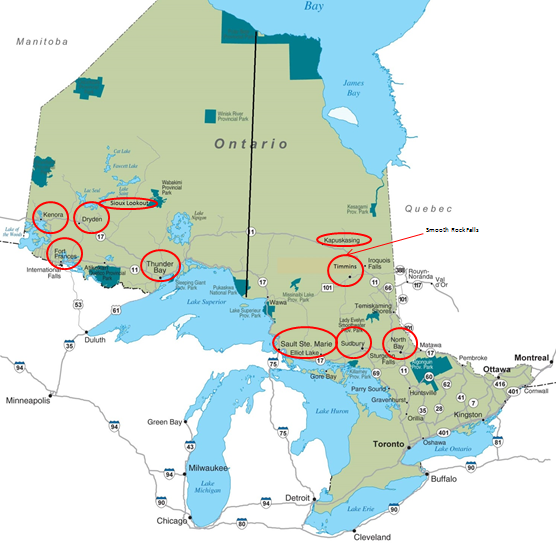

Supplement: S2 Appendix — (DOCX) [file pone.0225548.s002.docx]

## S3 Appendix

### Centre for Addiction and Mental Health (CAMH) Mobile Research Lab


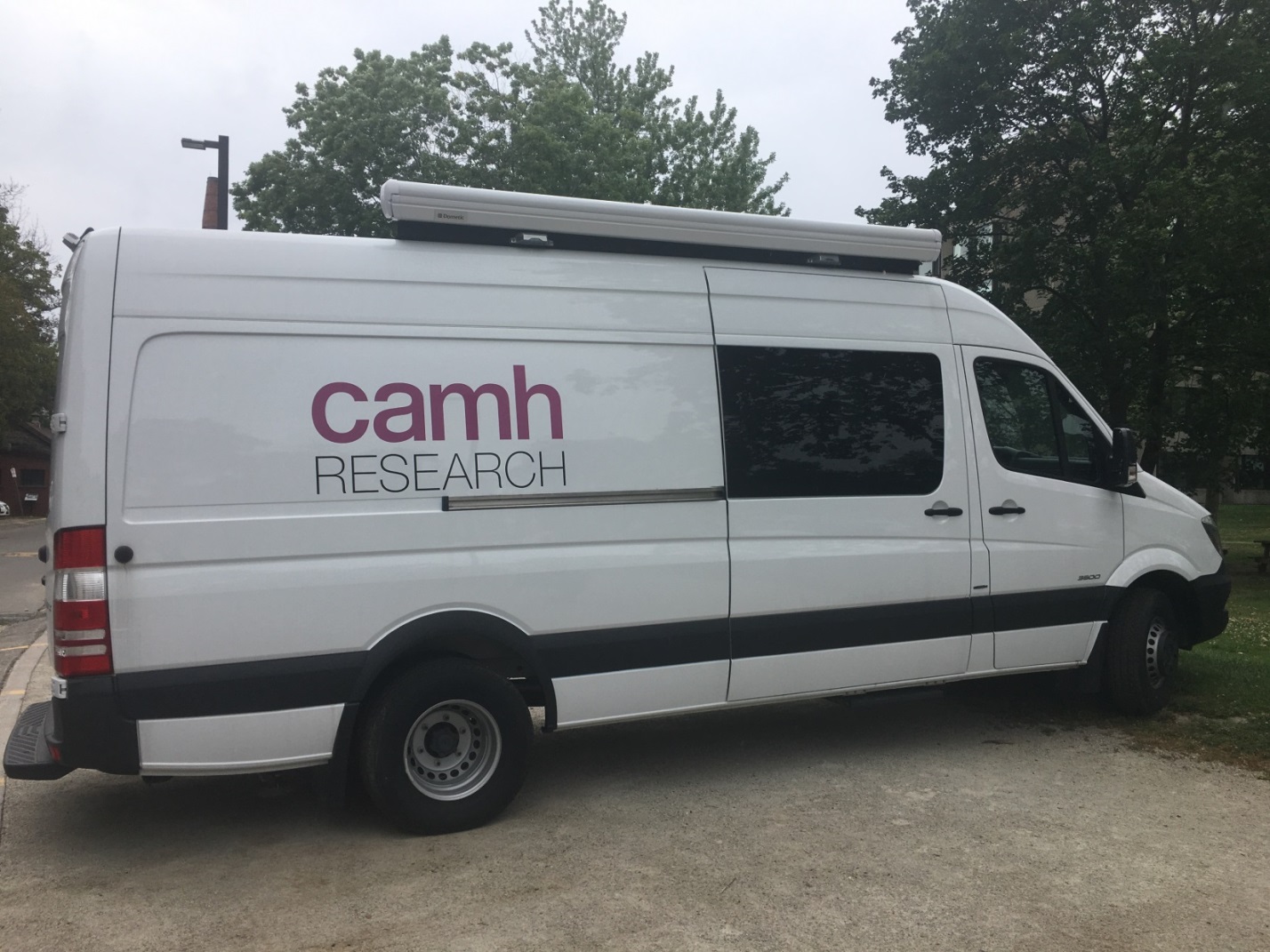

Supplement: S3 Appendix — (DOCX) [file pone.0225548.s003.docx]
